# Association Between Online Reviews of Substance Use Disorder Treatment Facilities and Drug-Induced Mortality Rates: Cross-Sectional Analysis

**DOI:** 10.2196/46317

**Published:** 2023-12-29

**Authors:** Matthew P Abrams, Raina M Merchant, Zachary F Meisel, Arthur P Pelullo, Sharath Chandra Guntuku, Anish K Agarwal

**Affiliations:** 1 Center for Digital Health University of Pennsylvania Perelman School of Medicine Philadelphia, PA United States; 2 Center for Emergency Care Policy and Research University of Pennsylvania Perelman School of Medicine Philadelphia, PA United States; 3 Department of Psychiatry University of California San Diego San Diego, CA United States; 4 Leonard Davis Institute of Health Economics University of Pennsylvania Philadelphia, PA United States; 5 Penn Injury Science Center University of Pennsylvania Philadelphia, PA United States; 6 Department of Computer and Information Science University of Pennsylvania Philadelphia, PA United States

**Keywords:** opioid use disorder, online reviews, drug-induced mortality, addiction, substance use disorder treatment, substance use disorder, patient-centered care, digital health, treatment, substance use, online review, drug use, mortality, database, addiction, detoxification, rehabilitation, communication, patient-centered

## Abstract

**Background:**

Drug-induced mortality across the United States has continued to rise. To date, there are limited measures to evaluate patient preferences and priorities regarding substance use disorder (SUD) treatment, and many patients do not have access to evidence-based treatment options. Patients and their families seeking SUD treatment may begin their search for an SUD treatment facility online, where they can find information about individual facilities, as well as a summary of patient-generated web-based reviews via popular platforms such as Google or Yelp. Web-based reviews of health care facilities may reflect information about factors associated with positive or negative patient satisfaction. The association between patient satisfaction with SUD treatment and drug-induced mortality is not well understood.

**Objective:**

The objective of this study was to examine the association between online review content of SUD treatment facilities and drug-induced state mortality.

**Methods:**

A cross-sectional analysis of online reviews and ratings of Substance Abuse and Mental Health Services Administration (SAMHSA)–designated SUD treatment facilities listed between September 2005 and October 2021 was conducted. The primary outcomes were (1) mean online rating of SUD treatment facilities from 1 star (worst) to 5 stars (best) and (2) average drug-induced mortality rates from the Centers for Disease Control and Prevention (CDC) WONDER Database (2006-2019). Clusters of words with differential frequencies within reviews were identified. A 3-level linear model was used to estimate the association between online review ratings and drug-induced mortality.

**Results:**

A total of 589 SAMHSA-designated facilities (n=9597 reviews) were included in this study. Drug-induced mortality was compared with the average. Approximately half (24/47, 51%) of states had below average (“low”) mortality rates (mean 13.40, SD 2.45 deaths per 100,000 people), and half (23/47, 49%) had above average (“high”) drug-induced mortality rates (mean 21.92, SD 3.69 deaths per 100,000 people). The top 5 themes associated with low drug-induced mortality included detoxification and addiction rehabilitation services (*r*=0.26), gratitude for recovery (*r*=–0.25), thankful for treatment (*r*=–0.32), caring staff and amazing experience (*r*=–0.23), and individualized recovery programs (*r*=–0.20). The top 5 themes associated with high mortality were care from doctors or providers (*r*=0.24), rude and insensitive care (*r*=0.23), medication and prescriptions (*r*=0.22), front desk and reception experience (*r*=0.22), and dissatisfaction with communication (*r*=0.21). In the multilevel linear model, a state with a 10 deaths per 100,000 people increase in mortality was associated with a 0.30 lower average Yelp rating (*P*=.005).

**Conclusions:**

Lower online ratings of SUD treatment facilities were associated with higher drug-induced mortality at the state level. Elements of patient experience may be associated with state-level mortality. Identified themes from online, organically derived patient content can inform efforts to improve high-quality and patient-centered SUD care.

## Introduction

Drug-induced mortality across the United States has continued to rise [1] from 6.2 to 21.6 age-adjusted deaths per 100,000 people over the last 20 years [2]. Recently, the Centers for Disease Control and Prevention (CDC) reported 70,630 drug overdose deaths in the United States—an average of 193 deaths every day [2]. People with substance use disorder (SUD) have higher prevalence rates of major medical conditions and a higher disease burden compared with the general population [3]. SUD-related morbidity and mortality are projected to increase over the next year [4]. There is an increased focus on ensuring that efforts to address and reduce drug-induced morbidity and mortality are patient centered to increase adoption [5,6].

To date, there are limited measures to evaluate patient preferences and priorities regarding SUD treatment [7,8], and many patients do not have access to evidence-based treatment options [9]. Patients and their families seeking SUD treatment may begin their search for an SUD treatment facility online, where they can find information about individual facilities, as well as a summary of patient-generated online reviews via popular platforms such as Google or Yelp [5]. While online reviews are not validated measures of quality of care as compared with Press Ganey or the Hospital Consumer Assessment of Healthcare Providers and Systems (HCAHPS), the use of online ratings of health care experiences continues to grow, reflecting the general trend of how consumers are seeking health-related information [10]. Prior studies of many medical settings, including essential health care facilities [11], mental health treatment facilities [12], hospitals [10], emergency departments [13], urgent care centers [13], and skilled nursing facilities, have demonstrated that online reviews may capture aspects of the patient experience that are associated with positive or negative ratings, as well as quality of care [14].

Online reviews of SUD treatment facilities may reflect information about factors associated with positive or negative patient satisfaction [15,16]. This content may provide insights to inform the development of SUD treatment performance metrics and patient-driven priorities. Evaluating this is important as understanding patient experiences is key to moving toward more patient-centered care and improved treatment services [17,18]. We sought to evaluate publicly available online reviews of US SUD treatment facilities to examine the association between online ratings of SUD treatment facilities and drug-induced mortality across the United States. We also aimed to explore if quality of care differences were reflected in reviews’ narrative content. We examined the association between thematic content of patient-generated online reviews associated with 1-star (lowest) versus 5-star (highest) ratings and drug-induced mortality.

## Methods

### Sample

All online reviews and ratings published on Yelp for outpatient SUD treatment facilities within the United States during September 2005 to October 2021 were collected. Facilities designated as non-SUD health facilities (eg, optometrists or retirement homes) were excluded (Multimedia Appendix 1). Consistent with prior studies on online reviews, analysis using natural language processes was used in SUD treatment facilities with 5 or more reviews [15].

We matched the list of US SUD treatment facilities to their corresponding facilities in the 2016 National Directory of Drug and Alcohol Abuse Treatment Facility Record published by the Substance Abuse and Mental Health Services Administration (SAMHSA). Matching was done using facility name and address to calculate the shortest string matching Levenshtein distance [19]. If an SUD treatment facility was not listed within the SAMHSA directory, then it was not included in the analysis.

Drug-induced mortality rates for each state were collected from the CDC WONDER Database from 2006 to 2019, and state averages were determined. Descriptive statistics were used to determine the univariate and bivariate distributions of Yelp review ratings and drug-induced mortality rates. Drug-induced mortality was treated as a continuous variable. States were considered to have “high” drug-induced mortality if their average drug-induced mortality rate was above the mean for all states. Likewise, states were considered to have “low” drug-induced mortality if their average drug-induced mortality rate was below the mean for all states.

### Generating Topics, Identifying Themes, and Examining Associations With Facility Online Ratings

Latent Dirichlet allocation (LDA) is a machine learning approach that groups co-occurring words into topics. These topics are then hand-coded to identify associated themes [20]. LDA uses an unsupervised dimension reduction procedure [20] to identify latent topics among large quantities of text. The distribution of LDA topics was extracted for each facility. Themes were categorized by an independent review by 2 members of the research team (AKA and MPA), and differences were reconciled by a third member (RMM).

Ordinary least-squares regressions were performed to generate topics associated with the drug-induced mortality rates of each facility’s state average. Pearson *r* was used to calculate effect size. For each topic generated, 10 reviews were identified. Specifically, the probability of all topics for each review was calculated, and subsequently, reviews that had the highest probability for each topic were identified. These 10 reviews were used by 3 coders (AKA, RMM, and MPA) to assign each topic a theme. The Benjamini-Hochberg *P* correction and *P*<.05 were used to identify significant correlations. Paired 2-tailed *t* tests with the Benjamini-Hochberg *P* correction were used to measure statistically significant associations between themes and state-level drug-induced mortality rates.

### Multilevel Modeling of the Association Between Yelp Ratings and Drug-Induced Mortality Rates

Because the multilevel mixed-effects linear regression model accounts for variation at the facility level, all states with facilities with at least 5 online ratings were included (n=51 states). We used null random-intercepts models to calculate intraclass correlations and variance partitioning coefficients to determine the degree of clustering in ratings at the facility and state levels. The average correlation of ratings in the same state (ie, intraclass correlation) was 0.03, while that among ratings from the same facility was 0.21. Variance components analysis showed that 2% of the variance in ratings was explained at the state level, 17% was explained at the facility level, and the remaining 81% was within facilities.

Likelihood ratio tests revealed that models that accounted for clustering at both the facility and state level fit the data better than those that accounted for only the former (*χ*^2^_1_=137.7, *P*<.001), only the latter (*χ*^2^_1_=946.5, *P*<.001), or neither (*χ*^2^_1_=1405.1, *P*<.001). Neither of the models that allowed the relationship between drug mortality and rating to vary at the state or facility level converged, so we proceeded with a 3-level, random-intercepts model with ratings nested in facilities and nested in states.

The 3-level, random-intercepts model used to assess the relationship between online review ratings and drug mortality rates integrated only 1 state-level predictor (drug-induced mortality rates), which was grand mean centered to improve interpretability of the intercept. As the outcome was on a 5-point Likert scale, we conducted a sensitivity analysis rerunning the model using a mixed-effects ordinal regression to see if it altered the results. There were no missing data for the predictor and outcome. All analyses were conducted in Stata (version 15; StataCorp).

### Ethical Considerations

This study was considered exempt by the University of Pennsylvania institutional review board.

## Results

### Descriptive Statistics of Sample

A total of 589 SUD treatment facilities listed within the SAMHSA directory (6.5% of 9061 US SAMSHA-designated facilities) met the inclusion criteria of having at least 5 reviews (n=9597 reviews; n=9597 ratings). These facilities belonged to 47 states. Most facilities represented the West US census region (n=316), followed by the South (n=130), Midwest (n=67), and Northeast (n=62). The number of online ratings of SUD treatment facilities was the same as the number of online reviews (ie, each online review had a corresponding rating, so the sample included 9597 reviews and 9597 ratings).

Ratings for the 589 facilities had a bimodal distribution with peaks at a rating of 1 (n=4546) and 5 (n=3649) with a median (IQR) of 2 (2-4). The mean (SD) facility rating was 2.82 (1.87). Among these facilities, the mean (SD) state-level drug-induced mortality rate was 17.57 (5.30; range 7.54-35.01) age-adjusted deaths per 100,000 people. States were considered to have “higher than average” (ie, “high”) or “lower than average” (ie, “low”) drug-induced mortality if their average drug-induced mortality rate was above or below the average of 17.57 age-adjusted deaths per 100,000 people.

### States With Low Drug-Induced Mortality Rates

A total of 24 (51%) of 47 states in our sample had a low drug-induced mortality rate (mean 13.40, SD 2.45 age-adjusted deaths per 100,000 people; see Table 1 for descriptive statistics for low and high drug-induced mortality states).

Tables 2 and 3 display themes, correlation coefficient, and example quotations for each theme from online reviews associated with high or low drug mortality rates. We identified 9 distinct themes associated with low drug mortality rates and 14 distinct themes associated with high drug mortality rates. The top 5 themes most correlated with a low mortality rate included the following: detox and addiction rehabilitation services (*r*=–0.26), gratitude for sobriety and recovery (*r*=–0.25), thankful for treatment (*r*=–0.25), caring staff and amazing experience (*r*=–0.23), and individualized recovery programs (*r*=–0.20; Tables 2 and 3). Review language correlated with positive or negative state-level drug mortality rates is displayed in Multimedia Appendix 2.

**Table 1:** Descriptive statistics for low and high drug-induced mortality states included in natural language processing analyses (n=47).

Category	SAMHSA^a^ facilities, n	Reviews, n	Reviews per facility, mean (SD)	Facility rating, mean (SD)
Low drug-induced mortality states^b^ (n=24)	399	6853	16.03 (20.40)	3.06 (1.11)
High drug-induced mortality states^b^ (n=23)	190	2744	13.24 (13.13)	2.64 (1.01)

^a^SAMHSA: Substance Abuse and Mental Health Services Administration.

^b^States with 5 or more reviews were included in the natural language processing analyses.

**Table 2:** Themes across substance use disorder facilities most associated with states with low drug-induced mortality rates^a^.

Theme	Drug mortality rates, Pearson *r* (95% CI)	Top words	Example reviews (redacted to maintain anonymity)
Detox and addiction rehabilitation services	–0.26 (–0.33 to –0.18)	Program, sober, recovery, detox, addiction, rehab, drug, alcohol, clean, living, house, drugs, new, meetings, step	“[Facility name] changed my life! I learned about the disease of addiction and how to cope with life without the use of drugs or alcohol in this program. I couldn't be more grateful for my [Facility name] family and I continue to live a life free of drugs and alcohol by working on myself in a twelve step program. Thank you for helping me discover a better way of living.”
Gratitude for sobriety and recovery	–0.25 (–0.32 to –0.17)	Life, am, sober, years, house, grateful, today, addiction, hope, myself, saved, live, helped, learned, gave	“Almost 3 years ago I moved into the [facility name]. I’ve been clean and sober ever since. The [facility name] gave me the structure and spiritual tools to learn how to live a life of meaning and how to be a contributing member of society sober! In July I'll celebrate 3 years continuous years of recovery and I owe my life to the 12 step program I work and the [facility name]. Thanks for everything [staff name].
Thankful for treatment	–0.25 (–0.32 to –0.17)	Life, thank, amazing, love, truly, helping, god, grateful, enough, helped, saved, beyond, special, heart, open	“I am truly grateful for the care I received at the [facility name]...support staff, dietary, counselors, therapy as well as amazing facilitators. I am both humbled and grateful for my newfound sobriety. I truly hope I can carry this message to help others.
Caring staff and amazing experience	–0.23 (–0.30 to –0.15)	Recommend, recovery, house, amazing, great, highly, best, beautiful, anyone, food, truly, clients, detox, caring, comfortable	“Amazing, clean facility. Caring staff, exceptional chefs. I highly recommend it. The detox house and residential house are extremely nice. The rooms are spacious and all amenities are provided.” “This place is absolutely amazing”
Individualized recovery programs	–0.20 (–0.27 to –0.12)	Program, recovery, treatment, addiction, support, clients, group, programs, individual, approach, environment, highly, team, each, sobriety	“My time spent at bayside marin has been life changing...[facility name] has given me the tools for successful recovery. The team is top notch - highly educated in the evolving field of recovery...The treatment is smart and individualized. They also offer a free alumni meeting one evening a week, which I attend often. It's a great aftercare resource.” “The staff at [facility name] is so caring, knowledgeable and professional. Their philosophy and addiction recovery model is progressive and holistic, treating the whole person and helping them relearn how to “do life” sober and happy...” “Facility Name” has a great vision for recovery. One size does not fit all. Finding a unique and individualized recovery path can mean the difference between temporarily quitting and truly experiencing life change.”
Appreciation of care team	–0.19 (–0.26 to –0.10)	Life, center, recovery, helped, best, recommend, amazing, truly, highly, team, love, grateful, saved, caring, hope	“I don’t have the words to express the depth of my gratitude for [facility name] and all the staff! I have an entirely different perspective on my whole life, and a clear understanding of myself, in a universal sense. I’m home, living my life in a light of love and compassion, thanks to the work we did at [facility name].” “I am extremely grateful for the experience and treatment I received at [facility name]. All of the staff and therapists are extremely caring and knowledgeable. In particular [name]. He was extremely important and influential in my treatment there. My family and I will be forever grateful for him and the rest of the staff at [facility name]. If you know anyone struggling with addiction, I would highly recommend [facility name].”
Group therapy sessions	–0.14 (–0.22 to –0.06)	Therapy, day, group, week, groups, meetings, therapist, sessions, classes, once, etc, class, aa, meeting, each	“I thought this class was going to be boring like most classes are. It was quite the opposite. [The class] was very interesting and educational… The instruction was awesome”
Clinic management	–0.12 (–0.20 to –0.04)	Clients, client, director, management, clinical, run, high, business, lack, completely, employees, poor, focus, communication, field	“[Facility name] has had a complete reboot with leadership, programming, and staff since mid-2015. They have achieved the coveted joint commission accreditation, and hired all new credentialed therapists as well as highly trained behavioral health techs. This program in no way resembles the decision point of the past which is a real source of pride with the staff and clients. The biggest change clients will see is in the expanded programming and activities covering seven days per week.”
Case management and legal support	–0.10 (–0.18 to –0.02)	Case, manager, court, classes, class, managers, legal, client, jail, course, huge, dui, problems, ordered	“something must be said about the love I received from this program that was above and beyond that which is the norm... the legal team (specifically Dr. [name]) rendered support with progress reports to the court throughout my legal proceedings, appeared in court for me and successfully got my 7 year prison sentence suspended with alternate sentencing to where I did no jail or prison time. I can’t begin to say how grateful I am to have her support along with the entire staff of [facility name]”.

^a^Significance was measured using a paired 2-tailed *t* test with the Benjamini-Hochberg *P* correction (*P*<.05).

**Table 3:** Themes across substance use disorder facilities most associated with states with high drug-induced mortality rates^a^.

Theme	Drug mortality rates, Pearson *r* (95% CI)	Top words	Example reviews (redacted to maintain anonymity)
Dissatisfaction with length of stay and discharge process	0.11 (0.03-0.19)	Facility, days, stay, discharge, hours, without, given, during, social, worker, plan, once, friend, upon, case	“Ugh! Horrible! Rude staff, no individual therapy, ridiculous rules! And discharge planning? What discharge planning? You’re on your own there.” “Complete lack of discharge planning. My daughter was sent home with no follow up care plan and they wouldn’t even ship her belongings.”
Insurance, payments, and billing	0.11 (0.03-0.19)	Insurance, pay, money, bill, billing, paid, company, payment, charged, received, financial, charge, covered, check, card	“Do not come here – they are the worst clinic. The doctors are fine but they have you waiting forever, they screwed up our billing and wanted us to pay over $1000 in bills they submitted to the wrong insurance company and then double billed them. Save your time and go to some other place.” “Misleading insurance coverage information – abundantly clear to me that they really only want private pay patients.” “Just received the bill for 6 days of nothing....$10,000.00 ’'m telling you to stay away from this nasty dirty place. absolutely worthless!!” “Questionable billing practices at [facility name]. My husband received not one but two bills totaling over $23,000.00. We discovered that neither bill had been submitted to insurance for payment prior to billing him directly for the full amounts.”
Therapy for co-occurring mental health disorders	0.12 (0.04-0.20)	Mental, therapy, therapist, depression, disorder, psychiatrist, health, eating, anxiety, diagnosis, inpatient, group, outpatient, disorders, social	“[Facility name] saved my life. I am very pleased with everything. I would recommend [Facility name] to anyone with eating disorders and mental health issues.” “Admitted for my eating disorder. Excellent physicians (especially Dr. [name]), therapists, nutritionists (specifically qualified for ED), nurses, and mental health workers. Always available to assist...They mostly use CBT (cognitive behavioral therapy) which is an effective method for all types of addictions/disorders...I’m glad...now I have the tools I need for my recovery...I highly recommend this treatment facility for patients with alcohol addiction, drug addiction, mental health disorders, and eating disorders.”
Mental health resources	0.14 (0.06-0.22)	Help, health, mental, need, issues, those, services, crisis, may, illness, willing, seek, substance, serious, deal	“Addiction and behavioral health issues are complex and serious. I have experienced ttc as a thorough and caring approach to improving the lives of people who ask for help.” “Very good with their counseling and resources for help.” “Caring group of mental health experts.” “Professional, kind, compassionate mental health services.”
Communication with nurse	0.16 (0.08-0.24)	Told, said, didn’t, then, got, nurse, left, asked, came, took, down, couldn’t, mom, let, saying	“I never once saw my nurse after being in the room for an hour. They were too busy gossiping at the nurses station so for the reviews 9 months ago that had a response from the hospital saying they'll work on it is BS they'll still prioritize talking instead of taking care of patients”
Patients feeling restrained or held against their will	0.17 (0.08-0.24)	Patients, down, leave, please, keep, hold, unit, send, prison, against, worse, police, sleep, admitted, allowed	“Awful awful place. do not go here. Go to your regular psych or doctor before you ever step foot in this institution. It is more like a prison than a mental health facility.” “A prison-like “health” facility where you may come out of a worse...wonder why the only place with open beds and kinda warned by medical hospital staff some patients without prison mentality or if they're not not totally insane please watch your backs especially vulnerable and young”
Patient complaints and privacy concerns	0.17 (0.09-0.25)	Patient, information, complaint, state, against, due, name, records, refused, privacy, report, unprofessional, file, director, law	“I recommend against using this company. I went for an assessment on my own accord and paid myself. The final report issued had many errors that they refused to correct...Giving a false assessment and not correcting it after the errors were pointed out. I recommend you go somewhere more professional.” “If I could give no stars I would. Hipaa violations, unethical, incorrect medications, unprofessional and beyond belief still in business. Buyer beware. My records were altered and I have to get them legally rectified...Copious violations.”
Communication regarding appointments and office closures	0.21 (0.13-0.28)	Told, said, called, asked, then, see, needed, next, until, pm, morning, friday, monday, today, hour	“It would have been nice if someone had told us you guys closed early today!!!!! My husband had an appointment at 4:20pm and when we got to the clinic at 4pm, security said it was closed!!!!! His appointment is not until 4:20pm. I called the call center and even they said the clinic closed at 5pm!!!!! We wasted our money for parking and most importantly our time!!!!!!” “Awful experience! Zero stars if possible!!! Their intake hours are supposed to be Monday through Friday 8:30am to 2pm. I was told by the [curse word] that answered the phones that the intake appointments take 1-2 hours. I went there at 1:50pm...They said it was too late and to come back tomorrow. How could it be too late? Apparently, the intake appointments are 2-4 hours now. They have a new system. yeah, a new waste of my time system. No thanks.”
Wait time for appointments	0.21 (0.13-0.28)	Appointment, time, minutes, wait, office, waiting, appointments, hour, before, late, scheduled, schedule, long, waited, seen	“The [facility name] go can shove it- if you arrive 5 minutes late- they tell you to go away. If you want an appointment you're looking months out- but show up to your appointment 20 minutes early- and you'll be waiting an hour after your appointment time...” “Appointment time - 12:30pm, arrival time - 12:15pm, current time - 1:39pm and I'm still waiting!! Because this place is located in a predominantly black and hispanic neighborhood, these people think they can disrespect our time and have us waiting here for over an hour!! Stay away!” “Very poorly managed time wise. My first appointment was over two hours late from the scheduled time. huge co-pay. My second appointment was also over an hour late even though it was maybe a 10 minute consultation. My yet to be third appointment has been rescheduled twice, once 15 minutes beforehand.”
(Dissatisfaction with) phone calls and lines of communication	0.21 (0.13-0.29)	Call, phone, called, back, calls, someone, left, times, number, calling, message, answer, messages, speak, hold	“No one answers the phone or calls you back. I can't get a prescription refilled. The automated system is like the people...doesn’t work. Don't waste your time.” “This company, after an initial consultation and intent to become a patient, did not respond to my multiple emails and several calls and voicemails for over 2 months.”
Front desk and reception experience	0.22 (0.14-0.29)	Rude, front, desk, treated, unprofessional, attitude, service, extremely, woman, worst, horrible, speak, lady, ask, name	“Extremely rude and unhelpful. When I called to make an appointment the therapist was dry, rude and clearly uninterested.” “The medical center is great and the staff however the front desk woman is extremely rude,cold and disrespectful. It's a shame to have someone like her representing [facility name].
Medication choices and prescription refills	0.22 (0.14-0.30)	Medication, meds, doctor, medications, off, drug, psychiatrist, prescription, prescribed, pain, anxiety, drugs, withdrawal, med, effects	“This office is unhelpful and apathetic about refilling prescriptions. I am on week two now of daily phone calls to get a non-narcotic antidepressant prescription refilled. There is no reason why it shouldn't be filled, yet my calls remain unreturned and the soonest I can see a doctor is three weeks despite having 0 days left of my meds, which they know.” “The standard of care is dismally low. Gaslighting by doctors, patients being told to go OD so they can qualify for care, and patients being put out addicted to a cocktail of pills without informed consent regarding withdrawal effects or tapering regimens. This place exists to make money, not to heal.” “The psychiatrist hastily prescribed a narcotic that had a negative interaction with my other medication, she ignored the list of meds.”
Rude and insensitive care	0.23 (0.15-0.31)	Go, here, don’t, give, worst, ever, horrible, stars, rude, anyone, star, zero, nothing, please, worse	“The nurses were horrible, unattentive, with no compassion whatsoever. Worst hospital experience ever. Wow! I hope I never have to go there again.” “Liars liars liars! incompetent, obnoxious apathetic, rude, arrogant. Seriously, the worst of humanity works here.”

^a^Significance was measured using a paired 2-tailed *t* test with the Benjamini-Hochberg *P* correction (*P*<.05).

### States With High Drug-Induced Mortality Rates

A total of 23 (49%) of 47 states in our sample had a high drug-induced mortality rate (mean 21.92, SD 3.69 age-adjusted deaths per 100,000 people; Table 1).

The top 5 themes most correlated with high drug mortality rates included care from doctors or providers (*r*=0.24), rude and insensitive care (*r*=0.23), medication choices and prescription refills (*r*=0.22), front desk and reception experience (*r*=0.22), and (dissatisfaction with) phone calls and lines of communication (*r*=0.21; Tables 2 and 3)*.*

### Associations Between Review Ratings and Drug-Induced Mortality Rates

Across all states (n=11, 941 ratings), the mean (SD) mortality rate was 17.1 (5.5; range 6.8-35.0) age-adjusted deaths per 100,000 people. Multilevel modeling revealed that in a typical facility in a state with an average drug mortality rate, the predicted average Yelp rating was 2.6 (95% CI 2.5-2.8) out of 5. On average, there was a negative association between drug mortality rate and Yelp ratings (b=–0.03, 95% CI –0.05 to –0.01; *P*=.005). Therefore, a state with a 10 deaths per 100,000 people increase in drug-induced mortality was associated with a 0.30 points lower average Yelp rating. This negative association was replicated in the mixed-effects ordinal regression model (b=–0.04, 95% CI –0.07 to –0.01, *P*=.004).

## Discussion

### Principal Findings

This study analyzed the association between online ratings and narrative review content from online reviews of US SUD treatment facilities and drug-induced mortality data from the CDC. The study has 2 main findings. First, we found that the average negative online ratings of SUD treatment facilities were associated with higher drug-induced mortality. Second, there were marked differences in the themes expressed between high versus low mortality states. These findings provide insights about the gap that persists in understanding the associations between online reviews and drug-induced mortality outcomes. Further, these results may help amplify patient-generated perceptions of poor quality of SUD care that may contribute to increased drug-induced mortality.

For every 10 deaths per capita increase in drug-induced mortality, the Yelp rating is expected to be 0.3 points lower. This is important, as little research has been conducted to closely examine the association between the online ratings and morbidity and mortality outcomes in the context of SUD treatment [11]. Consistent with a prior report that found that higher online ratings of essential health care facilities were associated with lower mortality [11], our findings suggest that online ratings may serve as a proxy for some components of quality of care such as communication with patients or availability of evidence-based treatments. This work also provides evidence that tools such as ATLAS [21], a website developed to help patients find and compare SUD treatment facilities, may have value in guiding patients to care options that fit their needs and preferences.

Recently, the Shatterproof foundation developed National Principles of Care for addiction treatment, evidence-based practices to improve outcomes for individuals with SUD [22]. Themes associated with low mortality were consistent with these principles. For example, their second principle, “A personal plan for every patient,” matched the theme “individualized recovery programs.” This theme is also in line with a recent partnership between Shatterproof, the American Society of Addiction Medicine, and OpenBeds to create a free, 13-item assessment to determine what type of SUD treatment aligns best with each patient’s needs [23].

These findings provide insights into aspects of patient experience within SUD care that are often difficult to capture with numerical surveys including a focus on “caring staff” and “communication.” Themes associated with high mortality states often pertained to poor communication and low-quality or non–evidenced-based care. Many of these identified themes can guide areas of improvement regarding the delivery of patient-centered and high-quality care. The identified themes indicate aspects of the patient experience that may contribute to high and low state-level mortality. Ultimately, these results underscore a process to unify patients’ “digital voices” to improve and inform treatment for SUD.

### Limitations

This study has several limitations. Reviews in the sample represent a small proportion of a facility’s patients, and facilities included represent a very small proportion of the SUD treatment infrastructure. Further, online reviews may not be representative of the population seen at each facility because Yelp does not verify the identity of the user posting a rating or review. Therefore, the use of only Yelp reviews as a source of online ratings and reviews may limit the impact of our findings. Additionally, 4 states (including Washington DC) did not have SUD treatment facilities with more than 5 reviews, limiting conclusions that can be drawn about the association between themes in online ratings and mortality in those states. Further, consistent with previously published methods [10-13,15] to analyze thematic online review content, the analyses in this study were not stratified by year, which limits conclusions that can be drawn. Specifically, our data are limited by the fact that the distribution of ratings by year is slightly skewed toward later years when reviews of health centers on Yelp became more popular. Other limitations of this study include its retrospective design, selection bias, and responder bias. A final limitation is that due to our sample size, our analyses were limited to mortality data at the state level despite the fact that county-level mortality data are generally available, so we could not explore facility-level services or practices that may contribute to high drug mortality. If more reviews become available, a county-level analysis in the future may provide more granular results. Our team attempted to run a similar analysis at the county level, but the intersection of mortality data from CDC and review data from Yelp was very small. Likewise, there may be possible heterogeneity across SUD populations in different states that limits the impact of these findings, as well as differences in state-level investment in SUD care and responses to drug-induced mortality rates that vary depending on state-level priorities and budgetary restrictions. Although state policy likely is linked to mortality, state-level policy differences were not likely captured in the patient-generated online content analyzed in this study.

This study also has strengths. Online review platforms serve as an organic, democratizing, and accessible space for patients to document their care experiences with rich narratives. While reviews are not representative, Yelp uses software in place to filter out inappropriate or inaccurate reviews. Moreover, the anonymity of reviews may encourage patients to express the true realities of their experiences without fear that it will impact their care. Therefore, analyses of online review content can provide insights to improve patient experiences and treatment delivery that may not be captured by numerical surveys or patient experiences surveys where patients may be concerned that their anonymity is not protected.

### Conclusions

At the state level, mean negative online ratings of SUD treatment facilities were associated with higher drug-induced mortality. Additionally, unique narrative content themes were identified online reviews across states with low or high mortality. Online reviews of SUD treatment facilities provide an opportunity to investigate and understand elements of the patient experience, quality of care, and state level mortality. The themes generated from online, organically derived patient content can inform and improve patient-centered care for SUD treatment. Future efforts to integrate these themes into the development of an SUD treatment facility-based performance and quality measures for SUD treatment may help to further elucidate what aspects of patient care may promote or improve both patient satisfaction and drug-induced mortality.

## Appendix

Multimedia Appendix 1 Excluded facilities based on Yelp category label.

Multimedia Appendix 2 Words most associated with online reviews in states with (A) high and (B) low drug-induced mortality rates. Relative font size represents stronger correlation with high or low mortality. Increased frequency of word use is represented by darker shading.

## Abbreviations

CDC: Centers for Disease Control and Prevention
HCAHPS: Hospital Consumer Assessment of Healthcare Providers and Systems
LDA: latent Dirichlet allocation
SAMHSA: Substance Abuse and Mental Health Services Administration
SUD: substance use disorder

## Data Availability

The data sets generated during and/or analyzed during this study are available from the corresponding author on reasonable request.
