# Supplementary material for: Association Between Online Reviews of Substance Use Disorder Treatment Facilities and Drug-Induced Mortality Rates: Cross-Sectional Analysis
Source: JMIR AI. 2023 Dec 29;2:e46317. doi: 10.2196/46317 (PMC11041514; doi:10.2196/46317)
Supplement: Multimedia Appendix 1 [file ai_v2i1e46317_app1.docx]

**Multimedia Appendix 1.** Excluded facilities based on Yelp category label.

| **Yelp Categories** | **# Facilities** | **Yelp Categories** | **# Facilities** | **Yelp Categories** | **# Facilities** |
| --- | --- | --- | --- | --- | --- |
| c_and_mh | 983 | optometrists | 6 | conciergemedicine | 1 |
| rehabilitation_center | 779 | hypnosis | 5 | habilitativeservices | 1 |
| addictionmedicine | 349 | retirement_homes | 5 | alternativemedicine | 1 |
| medcenters | 206 | weightlosscenters | 5 | reiki | 1 |
| hospitals | 169 | laboratorytesting | 5 | surgeons | 1 |
| psychiatrists | 116 | diagnosticimaging | 4 | halfwayhouses | 1 |
| nonprofit | 83 | occupationaltherapy | 4 | divorce | 1 |
| psychologists | 45 | walkinclinics | 4 | faithbasedcpc | 1 |
| physicians | 40 | yoga | 4 | fingerprinting | 1 |
| familydr | 35 | publicservicesgovt | 3 | localservices | 1 |
| behavioranalysts | 30 | periodontists | 3 | highschools | 1 |
| generaldentistry | 27 | parentingclasses | 3 | professional | 1 |
| drugstores | 16 | opticians | 3 | general_litigation | 1 |
| pharmacy | 15 | midwives | 3 | dentalhygienists | 1 |
| internalmed | 15 | podiatrists | 3 | healthcoach | 1 |
| lifecoach | 14 | careercounseling | 3 | apartments | 1 |
| convenience | 14 | diagnosticservices | 3 | preventivemedicine | 1 |
| obgyn | 13 | personalcare | 3 | skillednursing | 1 |
| duischools | 12 | dentists | 2 | herbalshops | 1 |
| naturopathic | 12 | adoptionservices | 2 | trafficschools | 1 |
| pediatricians | 11 | oralsurgeons | 2 | cannabis_clinics | 1 |
| cosmeticdentists | 11 | employmentagencies | 2 | dialysisclinics | 1 |
| nutritionists | 11 | pediatric_dentists | 2 | recreation | 1 |
| health | 10 | neurologist | 2 | specialed | 1 |
| emergencyrooms | 10 | dermatology | 2 | nephrologists | 1 |
| urgent_care | 9 | adultedu | 2 | psychic_astrology | 1 |
| chiropractors | 9 | emergencymedicine | 2 | culturalcenter | 1 |
| massage_therapy | 8 | lactationservices | 1 | oncologist | 1 |
| painmanagement | 8 | meditationcenters | 1 | educationservices | 1 |
| physicaltherapy | 8 | opthamalogists | 1 | hydrotherapy | 1 |
| acupuncture | 7 | tuina | 1 | medicalspa | 1 |
| homehealthcare | 7 | elementaryschools | 1 | nursepractitioner | 1 |
| orthodontists | 7 | gastroenterologist | 1 | anesthesiologists | 1 |
| endodontists | 6 | colonics | 1 | osteopathicphysicians | 1 |
|  |  |  |  | earnosethroat | 1 |
