# Supplementary figures and images for: Association Between Online Reviews of Substance Use Disorder Treatment Facilities and Drug-Induced Mortality Rates: Cross-Sectional Analysis
Source: JMIR AI. 2023 Dec 29;2:e46317. doi: 10.2196/46317 (PMC11041514; doi:10.2196/46317)

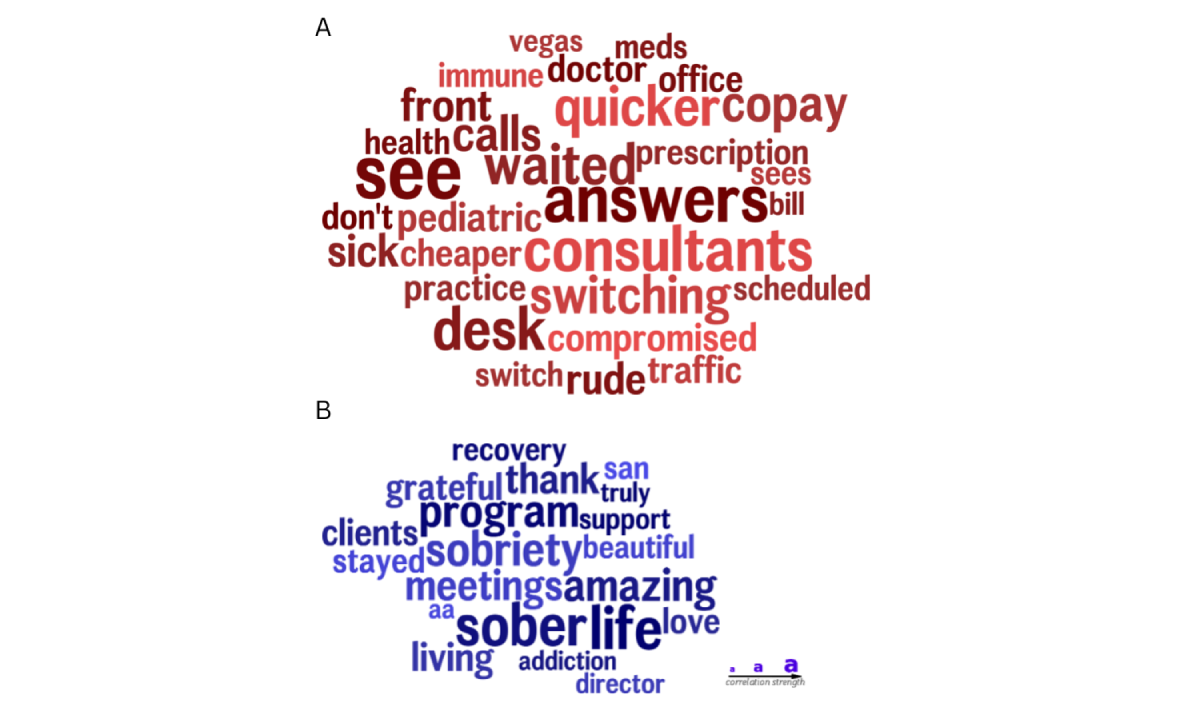

Supplement: Multimedia Appendix 2 [file ai_v2i1e46317_app2.png]
